# Methylation analysis of plasma DNA informs etiologies of Epstein-Barr virus-associated diseases

**DOI:** 10.1038/s41467-019-11226-5

**Published:** 2019-07-22

**Authors:** W. K. Jacky Lam, Peiyong Jiang, K. C. Allen Chan, Wenlei Peng, Huimin Shang, Macy M. S. Heung, Suk Hang Cheng, Haiqiang Zhang, O. Y. Olivia Tse, Radha Raghupathy, Brigette B. Y. Ma, Edwin P. Hui, Anthony T. C. Chan, John K. S. Woo, Rossa W. K. Chiu, Y. M. Dennis Lo

**Affiliations:** 1 Li Ka Shing Institute of Health Sciences, The Chinese University of Hong Kong, Shatin, New Territories, Hong Kong SAR, China; 2 Department of Chemical Pathology, The Chinese University of Hong Kong, Prince of Wales Hospital, Shatin, New Territories, Hong Kong SAR, China; 3 State Key Laboratory of Translational Oncology, Sir Y.K. Pao Centre for Cancer, The Chinese University of Hong Kong, Shatin, New Territories, Hong Kong SAR, China; 4 Department of Otorhinolaryngology, Head and Neck Surgery, The Chinese University of Hong Kong, Prince of Wales Hospital, Shatin, New Territories, Hong Kong SAR, China; 5 Department of Clinical Oncology, The Chinese University of Hong Kong, Prince of Wales Hospital, Shatin, New Territories, Hong Kong SAR, China

**Keywords:** Tumour biomarkers, Cancer screening, DNA methylation, Head and neck cancer

## Abstract

Epstein-Barr virus (EBV) is associated with a number of diseases, including malignancies. Currently, it is not known whether patients with different EBV-associated diseases have different methylation profiles of circulating EBV DNA. Through whole-genome methylation analysis of plasma samples from patients with nasopharyngeal carcinoma (NPC), EBV-associated lymphoma and infectious mononucleosis, we demonstrate that EBV DNA methylation profiles exhibit a disease-associated pattern. This observation implies a significant potential for the development of methylation analysis of plasma EBV DNA for NPC diagnostics. We further analyse the plasma EBV DNA methylome of NPC and non-NPC subjects from a prospective screening cohort. Plasma EBV DNA fragments demonstrate differential methylation patterns between NPC and non-NPC subjects. Combining such differential methylation patterns with the fractional concentration (count) and size of plasma EBV DNA, population screening of NPC is performed with an improved positive predictive value of 35.1%, compared to a count- and size-based only protocol.

## Abstract (web summary)

Epstein-Barr virus (EBV) is associated with different malignant diseases and circulating EBV DNA is a biomarker for nasopharyngeal carcinoma (NPC). Here, the authors report that plasma EBV DNA methylation profiles show disease-associated patterns and can help to distinguish NPC and non-NPC subjects.

## Introduction

Epstein–Barr virus (EBV) is known as an oncogenic virus through its association with a number of malignancies of epithelial and haematological origins^1^, including nasopharyngeal carcinoma (NPC)^2,3^, Burkitt’s lymphoma, Hodgkin’s lymphoma, natural killer-T cell (NK-T cell) lymphoma and post-transplant lymphoproliferative disease. Because of such associations, circulating EBV DNA has been explored for its diagnostic role in different EBV-associated malignancies^4–9^. For NPC, plasma EBV DNA is a well-established biomarker for prognostication and monitoring for recurrence^10–12^. Its additional clinical utility for NPC screening of asymptomatic individuals was confirmed in a recent large-scale prospective study^13,14^. For other EBV-associated malignancies, plasma EBV DNA was also shown to have prognostic significance^8,15,16^. Recently, our group has identified different fragmentation patterns and size profiles of plasma EBV DNA between NPC and non-NPC subjects^17^. Based on this differentiating feature, we have developed a second-generation NPC screening test which could achieve a higher positive predictive value with only one testing. With better understanding of the molecular characteristics of plasma EBV DNA, it is possible that the diagnostic performance of plasma EBV DNA testing can be further enhanced.

EBV DNA methylation, as a key epigenetic characteristic, has been studied to understand EBV biology^18–22^ in different EBV-associated diseases. These studies on EBV DNA methylation have focused on the analysis of methylation status of specific gene regions (e.g., promoters or transcription start sites)^19,20,23^. In one study, Fernandez et al.^19^ analysed the methylation status of 77 amplicons bearing transcription start sites in the EBV genome in the tumour tissues or cell line samples of different EBV-associated diseases. They showed that most gene promoters were methylated in tumour tissues or cell line samples of both NPC and EBV-associated lymphoma, whereas these promoters were unmethylated in cell lines in which the virus was in the lytic replicative state.

Currently, it is not known whether patients with different EBV-associated diseases would have distinct methylation profiles of circulating EBV DNA. Hence, in the current study, we perform genome-wide methylation profiling of circulating EBV DNA in the plasma of patients with different EBV-associated diseases. We report that the viral genome-wide EBV DNA methylation profiles exhibit a disease-associated pattern among the different diseases under investigation. To illustrate the potential clinical utility of this observation, we perform methylation profiling of plasma EBV DNA of NPC and non-NPC subjects from a previous screening cohort^13^. It is particularly challenging to analyse the methylation profiles of plasma EBV DNA when its concentration is expected to be low in early-stage cancers. We demonstrate the differential methylation patterns between NPC and non-NPC subjects, which could be potentially used to differentiate the two groups in the context of screening.

## Results

### Methylation analysis of EBV-associated diseases samples

We performed genome-wide methylation profiling of plasma DNA from 15 patients with NPC, 9 patients with EBV-associated lymphoma (6 cases of extranodal NK-T cell lymphoma, 1 case of post-transplant lymphoproliferative disorder, 1 case of Hodgkin’s lymphoma and 1 case of Burkitt’s lymphoma) and 5 patients with infectious mononucleosis. The subject characteristics are shown in Supplementary Table 1. Targeted bisulfite sequencing of plasma DNA was performed with enrichment of EBV DNA molecules by capture probes which covered the entire EBV genome. The methylation profiles of plasma EBV DNA for all the cases were analysed.

**Fig. 1:**
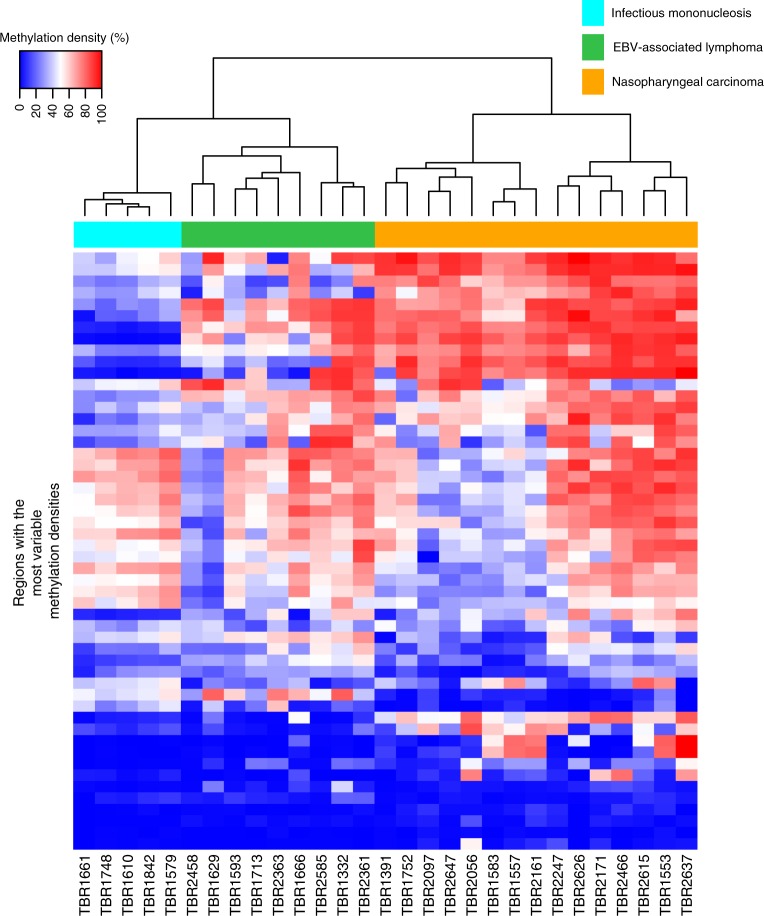
Distinctive plasma EBV DNA methylation profiles among different EBV-associated diseases. Unsupervised hierarchical clustering analysis of plasma EBV DNA methylome for the 29 samples from patients with different EBV-associated diseases. Each vertical bar represents one plasma sample. Each horizontal bar represents the selected 500-bp regions in the viral genome which demonstrated the most variable methylation densities (coefficient of variation > 30%) across all 29 samples. The corresponding methylation density of each region for all samples were represented in different colours. Samples of different patients with the same EBV-associated diseases were clustered together

Unsupervised hierarchical clustering analysis of the genome-wide methylation patterns of plasma EBV DNA was performed for all the 29 samples of EBV-associated diseases (Fig. 1). The clustering analysis was based on the regions in the viral genome which exhibited the most variable methylation densities across all the samples. Hence, the EBV genome was first divided into non-overlapping regions of 500 bp. The methylation densities of each 500 bp-region, defined as the percentage of methylated CpG sites out of all CpG sites for sequencing reads mapped to the regions, were calculated for all the samples. The methylation densities of these 500-bp regions were compared among different samples. Those regions with the most variable methylation densities among these 29 samples (coefficient of variation (i.e., standard deviation/mean) > 30%) were selected for the clustering analysis. Remarkably, plasma samples from different patients with the same EBV-associated disease were clustered together (Fig. 1). The segregation of NPC and infectious mononucleosis samples in the clustering analysis was confirmed with a permutation-based statistical test (pvclust package implemented in R). This suggested that methylation profiles of plasma EBV DNA were distinct among different EBV-associated diseases, and patients with the same disease would share a similar methylation pattern.

### Analysis of NPC and non-NPC subjects from a screening cohort

After identifying that distinctive plasma EBV DNA methylation patterns did exist amongst different EBV-associated diseases, we explored the potential clinical utility of such analyses in the context of cancer screening. Specifically, plasma EBV DNA analysis was recently validated for screening NPC^13^. In such a screening context, approximately 5% of the general population harbours detectable levels of EBV DNA in their plasma by polymerase chain reaction (PCR)-based testing on one occasion^4,24^. These individuals would contribute to the false-positive group. It would be ideal if the test method could differentiate NPC patients from the false-positive group of non-NPC subjects with detectable levels of plasma EBV DNA. Here, we explored if we could differentiate the NPC from the non-NPC group by methylation analysis of their plasma EBV DNA.

Archived plasma samples of NPC patients and non-NPC subjects with detectable plasma EBV DNA from the prospective screening trial^13^ were used to explore the clinical utility of methylation analysis of plasma EBV DNA. In the screening cohort of 20,174 asymptomatic male subjects, we identified 34 NPC patients and 1078 non-NPC subjects with detectable plasma EBV DNA at baseline by PCR-based testing. Since a two time-point testing protocol was adopted, these 1078 non-NPC subjects could be further classified into either a transiently positive group (positive at baseline and negative at the follow-up test) (*n* = 803) or a persistently positive group (positive at both the baseline and follow-up tests) (*n* = 275) based on their plasma EBV DNA test results.

In the current study, we first explored the differences in the methylation profiles of plasma EBV DNA between NPC and non-NPC subjects in an exploratory sample set. The exploratory set consisted of 10 NPC patients and 40 non-NPC subjects (20 with transiently positive and 20 with persistently positive plasma EBV DNA results) randomly selected from the previous screening cohort. We subsequently validated our findings in the validation sample set which consisted of the remaining 23 NPC patients and another 120 randomly selected non-NPC subjects (90 with transiently positive and 30 with persistently positive plasma EBV DNA results) from the screening cohort. One NPC patient was not analysed because the sample had been exhausted. The number of non-NPC subjects to be included in the validation set was based on a power analysis, which would be described in the Methods section. The baseline plasma samples collected at enrolment into the screening study would be analysed. We have also included another 14 NPC patients from an unscreened cohort in the validation sample set. These 14 NPC patients in the validation set did not overlap with those NPC patients used for mining the NPC-associated differentially methylated regions (DMRs), which would be described in the next section. The subject characteristics are shown in Table 1.

**Table 1:** Subject characteristics in the exploratory and validation sample sets

	Non-NPC subjects with transiently positive plasma EBV DNA in the exploratory set	Non-NPC subjects with persistently positive plasma EBV DNA in the exploratory set	NPC patients from the screening cohort in the exploratory set	Non-NPC subjects with transiently positive plasma EBV DNA in the validation set	Non-NPC subjects with persistently positive plasma EBV DNA in the validation set	NPC patients from the screening cohort in the validation set	NPC patients from an external cohort in the validation set
Number	20	20	10	90	30	23	14
*Sex*							
M	20	20	10	90	30	23	13
F	0	0	0	0	0	0	1
Median age, year (IQR)	53.5 (46–57)	48 (43–58)	52.5 (49–55)	54 (49–58)	54 (49–59)	52 (44.5–55)	57 (45.5–62)
*Tumour stage*							
I	NA	NA	5	NA	NA	10	2
II	NA	NA	1	NA	NA	7	0
III	NA	NA	4	NA	NA	4	5
IV	NA	NA	0	NA	NA	2	7

### NPC-associated DMRs

**Fig. 2:**
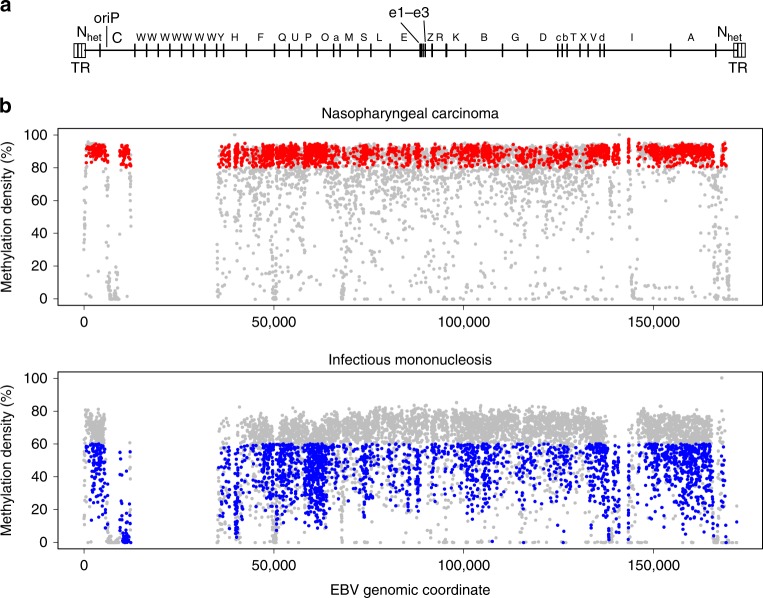
Mining of NPC-associated DMRs in the EBV genome. **a** *Bam*HI restriction map of the EBV genome is shown. **b** Methylation densities of CpG loci across the EBV DNA genome from the pooled sequencing data of the 15 cases of NPC and the pooled data of the 5 cases of infectious mononucleosis used for mining of DMRs. Each dot (grey and coloured) shows the methylation density at a corresponding CpG site. A DMR was constructed by two or more differentially methylated CpG sites (>80% in the pooled data of NPC and <60% in the pooled data of infectious mononucleosis) within 200 bp. The coloured dots highlight those CpG sites which fulfilled our criteria. The red dots are those CpG sites with the methylation densities greater than 80% in the pooled data of NPC and the blues dots are those with the methylation densities less than 60% in the pooled data of infectious mononucleosis. Viral DNA fragments mapped to the *Bam*HI-W repeat region presented ambiguities in alignment to the exact member of the repeat family. Hence, this region was not used in the final approach

We first identified the NPC-associated DMRs in the EBV genome. We pooled the sequencing data of all the 15 cases of NPC and 5 cases of infectious mononucleosis which we had analysed their plasma samples as described in Fig. 1. The methylation profiles of plasma EBV DNA of the two disease types were analysed and compared (Fig. 2b). The methylation densities, defined as the percentage of CpG sites being methylated, of all loci in the EBV genome were derived. A CpG site was defined as differentially methylated if the methylation density of the site was >80% in the pooled sequencing data of NPC and <60% in the pooled data of infectious mononucleosis. Such cutoffs were chosen based on the performances of the methylation-based analysis (described in the next section) in the exploratory sample set using different DMR sets derived from different cutoffs and the details are elaborated in the section “Methods”. The DMRs would include all differentiated methylated CpG sites. When two or more differentially methylated CpG sites were located within 200 bp, they were grouped together to form a DMR. With these selection criteria, we identified a total of 158 DMRs across the EBV genome. The genomic coordinates of these 158 DMRs were shown in the Supplementary Table 2.

To allow quantitative comparison of the methylation profiles of plasma EBV DNA between samples, we developed a metric, the EBV DNA methylation score, to represent the aggregated methylation levels for EBV DNA reads within the pre-defined NPC-associated DMRs. The methylation score was calculated by the following equation:

1 $$	\mathrm{EBV}\;\mathrm{DNA}\;\mathrm{methylation}\;\mathrm{score}\\ 	 = \frac{\mathrm{methylated}\;\mathrm{CpGs}}{\mathrm{methylated}\;\mathrm{CpGs} + \mathrm{unmethylated}\;\mathrm{CpGs}} \times 100.$$

The calculation of EBV DNA methylation score would be detailed in Methods and Supplementary Fig. 1.

### Methylation-based analysis in the exploratory sample set

**Fig. 3:**
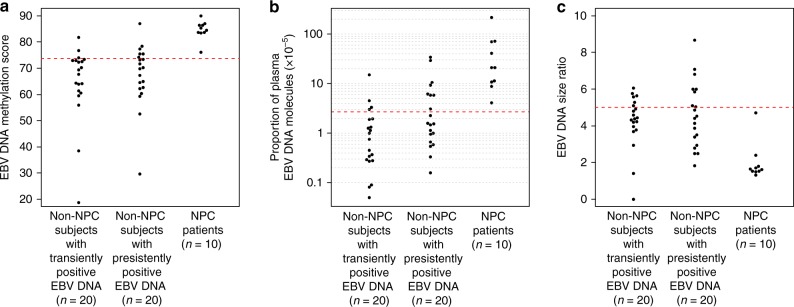
Methylation-, count- and size-based analyses of plasma EBV DNA in the exploratory sample set. **a** The EBV DNA methylation scores of the NPC patients and non-NPC subjects with transiently positive and persistently positive results are shown. A cutoff value in the EBV DNA methylation score was defined at 3 standard deviations below the mean of the methylation scores of these 10 NPC patients in the exploratory dataset. The cutoff value of 73.7 is denoted by the red dotted line. **b** The proportion of EBV DNA reads among the total number of sequenced plasma DNA reads (both human and viral) of the NPC patients and non-NPC subjects with transiently positive and persistently positive results are shown. A cutoff value in the proportion of plasma EBV DNA reads was defined at 3 standard deviations below the mean of the logarithmic values of portion of EBV DNA reads of the 10 NPC patients in the exploratory dataset. The cutoff value of 2.7 × 10^−5^ is denoted by the red dotted line. **c** The EBV DNA size ratios of the NPC patients and non-NPC subjects with transiently positive and persistently positive results are shown. A cutoff value was defined at 3 standard deviations above the mean values of the EBV size ratios of all the 10 NPC patients in the exploratory dataset. The cutoff value of 5.0 is denoted by the red dotted line. Source data are provided as a Source Data file

Targeted bisulfite sequencing was used to capture and analyse the EBV DNA in the archived plasma samples of NPC patients and non-NPC subjects in the exploratory set. We analysed the EBV DNA methylation score based on reads overlapped at the NPC-associated EBV DMRs for each plasma sample. The median EBV DNA methylation score was significantly higher in NPC patients (median = 84.9, IQR: 83.6–86.4) than non-NPC subjects with transiently positive (median = 68.5, IQR: 61.1–72.9) and persistently positive (median = 69.9, IQR: 62.6–74.5) EBV DNA results (Kruskal–Wallis test, *p* < 0.0001) (Fig. 3a). These results demonstrated that methylation-based analysis of plasma EBV DNA could be used to differentiate NPC from non-NPC subjects.

A cutoff value in the EBV DNA methylation score was defined at 3 standard deviations below the mean of the methylation scores of these 10 NPC patients in the exploratory dataset. The cutoff value of 73.7 was obtained (Fig. 3a). Using this cutoff value, 3 of the 20 subjects with transiently positive and 6 of the 20 subjects with persistently positive EBV DNA results and all NPC patients passed the cutoff in the isolated methylation-based analysis. The sensitivity and specificity at this cutoff for NPC detection were therefore 100% and 77.5%, respectively (Table 2). The individual methylation scores over the 158 DMRs for all the samples in the exploratory set were stated in Supplementary Data 1 for reference.

**Table 2:** Sensitivities and specificities of the isolated and combined analysis at the defined cutoffs in both exploratory and validation cohorts. The corresponding 95% confidence intervals are bracketed

		Sensitivity (%)	Specificity (%)
Exploratory cohort	Methylation based	100%	77.5% (62.5–87.7%)
	Count based	100%	70% (54.5–81.9%)
	Size based	100%	35% (22.1–50.5%)
	Combined methylation, count and size based	100%	87.5% (73.9–94.5%)
Validation cohort	Methylation based	100%	83.3% (75.7–88.9%)
	Count based	100%	75% (66.6–81.9%)
	Size based	100%	35.8% (27.8–44.7%)
	Combined methylation, count and size based	100%	94.2% (88.5–97.1%)

### Count- and size-based analysis in the exploratory sample set

Previously we had observed differences in the quantitative and size profiles of plasma EBV DNA between NPC patients and non-NPC subjects^17^. In the current study, we evaluated if similar observations could still be obtained after bisulfite treatment of plasma DNA. For the count-based analysis, we analysed the proportion of all EBV DNA reads among the total number of human and viral sequenced reads after removal of PCR duplicates. In the exploratory sample set, NPC patients (median = 2.1 × 10^−4^, IQR: 1.1 × 10^−4^ to 6.2 × 10^−4^) had a statistically higher proportion of EBV DNA reads than non-NPC subjects with transiently positive (median = 8.8 × 10^−6^, IQR: 2.9 × 10^−6^ to 1.9 × 10^−5^) and persistently positive results (median = 1.6 × 10^−5^, IQR: 8.9 × 10^−6^ to 5.9 × 10^−5^) (Kruskal–Wallis test, *p* < 0.0001) (Fig. 3b).

The size-based analysis was based on the observation of a characteristic nucleosomal size profile of plasma EBV DNA from NPC patients. Plasma EBV DNA from non-NPC subjects, on the contrary, exhibited a different size profile. As a result, NPC patients had a lower proportion of short EBV DNA molecules (less than 110 bp) when compared to the non-NPC subjects. From our current bisulfite sequencing data, the size profiles of plasma EBV DNA from an NPC patient and a non-NPC subject are shown in Supplementary Fig. 2. Previously, we had developed a metric, the EBV DNA size ratio, to measure the difference in the size profiles between the two groups. The EBV DNA size ratio was defined as the proportion of short EBV DNA fragments within the size range of 80–110 bp normalised by the proportion of autosomal fragments within the same size range

2 $$	\mathrm{EBV}\;\mathrm{DNA}\;\mathrm{size}\;\mathrm{ratio} \\ 	 = \frac{\mathrm{Proportion}\;\mathrm{of}\;\mathrm{EBV}\;\mathrm{DNA}\;\mathrm{within}\;80 - 110\;\mathrm{bp}}{\mathrm{Proportion}\;\mathrm{of}\;\mathrm{autosomal}\;\mathrm{DNA}\;\mathrm{within}\;80 - 110\;\mathrm{bp}}.$$

In the exploratory set, the median size ratio of the samples from NPC patients (median = 1.6, IQR: 1.5–1.8) was significantly lower than the median size ratios of samples from non-NPC subjects with transiently positive (median = 4.4, IQR: 3.9–5.1) and persistently positive results (median = 4.5, IQR: 3.3–5.9) (Kruskal–Wallis test, *p* < 0.0001) (Fig. 3c).

The proportions of EBV DNA reads and the EBV DNA size ratios for all the samples in the exploratory set were stated in Supplementary Data 1 for reference.

The count- and size-based analysis results of NPC patients from our current exploratory dataset confirmed similar findings of the molecular profiles of plasma EBV DNA reported previously in the non-bisulfite sequencing dataset^17^.

Similarly, based on the exploratory dataset, we defined cutoff values in the count-based and size-based analyses in order to achieve 100% detection sensitivity for all the 10 NPC cases. In the count-based analysis, a cutoff value in the proportion of plasma EBV DNA reads was defined at 3 standard deviations below the mean of the logarithmic values of portion of EBV DNA reads of the 10 NPC patients in the exploratory dataset. The cutoff value of 2.7 × 10^−5^ was obtained (Fig. 3b). At this cutoff value, 4 of the 20 subjects with transiently positive and 8 of the 20 subjects with persistently positive EBV DNA results and all NPC patients passed the cutoff, yielding a sensitivity of 100% and a specificity of 70% the isolated count-based analysis in the exploratory dataset (Table 2).

In the size-based analysis, a cutoff value in the EBV DNA size ratio was defined at 3 standard deviations above the mean values of the EBV DNA size ratios of the same 10 NPC patients. The cutoff value of 5.0 was obtained (Fig. 3c). At this cutoff value, 14 of the 20 subjects with transiently positive and 12 of the 20 subjects with persistently positive EBV DNA results and all NPC patients passed the cutoff. The calculated sensitivity of the isolated size-based analysis was 100% and the specificity was 35% at this cutoff (Table 2).

### Combined methylation-, count- and size-based analysis

In the combined methylation-, count- and size-based analysis, a plasma sample was flagged as positive if its sequencing data concurrently passed all the corresponding cutoffs in the three parameters. Using the cutoffs defined in the exploratory dataset, in the combined analysis, all the samples of NPC patients in the exploratory set could be captured. There were 0 (out of 20) subjects with transiently positive and 5 (out of 20) subjects with persistently positive results who passed both all the three cutoffs. The calculated sensitivity and specificity were therefore 100% and 87.5%, respectively at these cutoffs (Table 2).

The analytical definitions of the combined analysis and the cutoffs were locked down before validation.

### Validation of methylation-, count- and size-based analyses

**Fig. 4:**
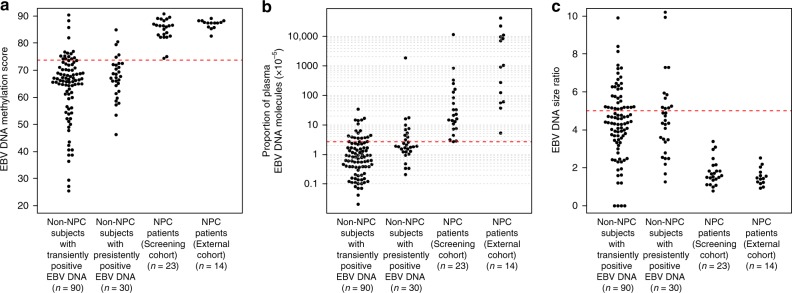
Methylation-, count- and size-based analyses of plasma EBV DNA in the validation sample set. The cutoffs in the corresponding analyses defined in the exploratory dataset are shown. **a** The EBV DNA methylation scores of the NPC patients (from both the screening and external cohorts) and non-NPC subjects with transiently positive and persistently positive results are shown. The same cutoff value of 73.7 defined in the exploratory dataset is denoted by the red dotted line. **b** The proportion of EBV DNA reads among the total number of sequenced plasma DNA reads (both human and viral) of the NPC patients (from both the screening and external cohorts) and non-NPC subjects with transiently positive and persistently positive results are shown. The same cutoff value of 2.7 × 10^−5^ defined in the exploratory dataset is denoted by the red dotted line. **c** The EBV DNA size ratios of the NPC patients (from both the screening and external cohorts) and non-NPC subjects with transiently positive and persistently positive results are shown. The same cutoff value of 5.0 defined in the exploratory dataset is denoted by the red dotted line. Source data are provided as a Source Data file

Plasma samples of NPC and non-NPC subjects in the validation set were analysed with targeted bisulfite sequencing using the same capture-probe set. For the methylation-based analysis, NPC patients from both the screening cohort (median = 86.3, IQR: 82.8–87.7) and the external cohort (median = 87.4, IQR: 86.3–88.1) had significantly higher EBV DNA methylation scores than non-NPC subjects with transiently positive (median = 66.3, IQR: 59.7–71.0) and persistently positive (median = 67.4, IQR: 62.7–72.1) results (Kruskal–Wallis test, *p* < 0.0001) (Fig. 4a). These results suggested that the finding of high EBV DNA methylation score, which implied hypermethylated EBV DNA fragments within NPC-associated DMRs, was not only limited to the screening cohort but was also observed in the external cohort of NPC patients. At the cutoff value of 73.7 defined in the exploratory dataset, all the NPC samples from both cohorts had EBV DNA methylation scores higher than the cutoff and could therefore be captured. There were 15 (out of 90) non-NPC subjects with transiently positive results and 5 (out of 30) subjects with persistently positive results who passed the cutoff in the methylation-based analysis (Fig. 4a). The calculated sensitivity of the isolated methylation-based analysis was 100% and the specificity was 83.3% at this cutoff (Table 2).

We performed a DMR down-sampling analysis and observed a decline in the area under the receiver operating characteristic (AUROC) values of the methylation-based analysis in NPC detection with serial down-sampling of DMRs (Supplementary Fig. 3). The DMR down-sampling analysis would be detailed in the section “Methods”.

In the current study, we adopted a protocol of employing a uniform input volume of 4 mL of plasma. Such a fixed-volume approach has been adopted by many groups for clinical applications of cell-free DNA analysis, such as for non-invasive prenatal testing^25–27^. As each ml of plasma could contain different amounts of DNA, the fixed-volume approach would result in different amounts of plasma DNA being sequenced for different cases. We observed a lower mean total plasma DNA concentration among the NPC cases from the screening cohort (5.5 ng/mL) than the non-NPC cases with transiently (10.1 ng/mL) or persistently positive plasma EBV DNA (8.2 ng/mL) (Kruskal–Wallis test, *p* < 0.0001) (Supplementary Fig. 4). There is no difference in the mean concentrations between the NPC cases from the external cohort (10.0 ng/mL) and the non-NPC cases (Kruskal–Wallis test, *p* = 0.86). Noting the different amounts of plasma DNA input for different cases, we performed a down-sampling analysis of the sequencing data for the NPC and non-NPC cases in the validation cohort to evaluate the potential impact of sequencing depth on the performance of the methylation-based analysis. The down-sampling analysis showed comparable performances of the methylation-based analysis based on equal number of down-sampled reads and that based on all fragments (Supplementary Fig. 5). This suggested that the methylation-based analysis could provide additional differentiating power of NPC from non-NPC samples independent of count-based analysis. The down-sampling analysis would be described in the section “Methods”.

For the count-based analysis, NPC patients from both the screening (median = 2.1 × 10^−4^, IQR: 9.1 × 10^−5^ to 1.1 × 10^−3^) and external (median: 9.7 × 10^−3^, IQR: 7.6 × 10^−4^ to 9.3 × 10^−2^) cohorts had significantly higher proportions of plasma EBV DNA reads than non-NPC subjects with transiently (median = 8.0 × 10^−6^, IQR: 3.7 × 10^−6^ to 2.3 × 10^−5^) and persistently positive (median = 1.9 × 10^−5^, IQR: 1.2 × 10^−5^ to 4.2 × 10^−5^) results (Kruskal–Wallis test, *p* < 0.0001) (Fig. 4b). All the NPC samples from both cohorts had the proportions of EBV DNA reads higher than the cutoff value of 2.7 × 10^−5^ defined in the exploratory dataset. There were 20 (out of 90) non-NPC subjects with transiently positive and 10 (out of 30) subjects with persistently positive results who passed the cutoff in the count-based analysis (Fig. 4b). The calculated sensitivity of the isolated count-based analysis was 100% and the specificity was 75% at this cutoff (Table 2).

For the size-based analysis, NPC patients from both the screening (median = 1.6, IQR: 1.4–2.0) and external (median = 1.4, IQR: 1.2–1.7) cohorts had significantly lower EBV DNA size ratios than non-NPC subjects with transiently (median = 4.4, IQR: 3.5–5.2) and persistently positive (median = 4.4, IQR: 3.4–5.2) results (Kruskal–Wallis test, *p* < 0.0001) (Fig. 4c). With the cutoff value of 5.0 defined in the exploratory dataset, all the NPC samples from both cohorts had the size ratios lower than the cutoff, and 58 (out of 90) non-NPC subjects with transiently positive and 19 (out of 30) subjects with persistently positive results passed the cutoff (Fig. 4c). The calculated sensitivity of the isolated size-based analysis was 100% and the specificity was 35.8% at this cutoff (Table 2). The EBV DNA methylation scores, proportion of EBV DNA reads and EBV DNA size ratios for all the samples in the validation set were stated in Supplementary Data 1 for reference.

### Combined analysis in the validation sample set

Using the cutoffs defined in the exploratory dataset, in the combined methylation-, count- and size-based analysis, all the samples of NPC patients in the validation set could be captured. There were 4 (out of 90) subjects with transiently positive and 3 (out of 30) subjects with persistently positive results who passed all the three cutoffs. The sensitivity and specificity of the combined analysis in the validation set were calculated to be 100% and 94.2%, respectively (Table 2).

### Modelled performance in the screening cohort

The above data indicated that the best screening performance for NPC could be obtained from the simultaneous analysis of the methylation, quantitative and size profiles of plasma EBV DNA. While the real-time PCR-based detection of plasma EBV DNA achieved a high negative predictive value, this combined methylation-, count- and size-based analysis would best serve as a confirmatory test to differentiate NPC from non-NPC subjects with detectable EBV DNA by PCR-based analysis. A plasma sample was considered as test-positive in the combined analysis if it simultaneously passed the methylation-, count- and size-based cutoffs. We modelled the diagnostic performance of incorporation of this combined analysis in the entire set of our previously reported screening cohort^13^ based on the performance in the validation set. Only the NPC and non-NPC samples from the screening cohort, but not the external cohort, were included for the modelling. In the validation cohort, at cutoffs defined in the exploratory set, 4 out of 90 (4.4%) non-NPC subjects with transiently positive, 3 out of 30 (10%) subjects with persistently positive and all 23 (100%) NPC patients passed the three cutoffs in the combined analysis. When taken into account that one subject with undetectable plasma EBV DNA by PCR-based testing developed NPC within 1 year, the sensitivity of the current protocol would be 97.1%. The estimated number of subjects with false-positive test results from the combined analysis was 63 (4.4% × 803 (transiently positive group) + 10% × 275 (persistently positive group)). The estimated specificity would be 99.7%. The corresponding PPV and false-positive rate would be 35.1% and 0.31%. The modelled performance of the methylation-, count-, size-based and combined analysis is shown in Table 3. Of note, there is a substantial increase in PPV with addition of the methylation-based analysis to the combined analysis involving count- and size-based analyses only.

**Table 3:** Modelled performance of the methylation-, count-, size- and combined analyses in the screening cohort by target-capture bisulfite sequencing

	Sensitivity (%)	Specificity (%)	PPV (%)
Methylation based	97.1	99.1	15.9
Count based	97.1	98.7	11.2
Size based	97.1	96.6	4.7
Combined count and size based	97.1	99.1	16.6
Combined methylation, count and size based	97.1	99.7	35.1

## Discussion

Our current study reports the genome-wide methylation profiles of plasma EBV DNA in NPC, EBV-associated lymphoma and infectious mononucleosis. In our clustering analysis of plasma EBV DNA methylomes, we observed that samples from different patients within the same group of EBV-associated disease were clustered together and yet they were more distant from samples of different classes of diseases. These results implied that distinctive plasma EBV DNA methylation profiles existed among the different diseases that we have investigated. We then explored the clinical potential of such an observation and developed the methylation-based analysis of plasma EBV DNA and showed that it improved the specificity of EBV DNA-based NPC screening test.

Using the archived plasma samples from the previous screening cohort, the methylation-based analysis of plasma EBV DNA was shown to achieve a high specificity of NPC identification at an observed sensitivity of 100%. The high diagnostic performance of the methylation-based analysis was contributed by the use of sequencing-based analysis and the selection of NPC-associated DMRs. Bisulfite sequencing allowed simultaneous analysis of a multitude of methylation markers across the whole viral genome. This is in contrast to the analysis based on a single methylation marker which might theoretically result in lower sensitivity for detection of cancer. This is supported by the result of our DMR down-sampling analysis, in which we have observed a decline in the AUROC values of the methylation-based analysis in NPC detection with serial down-sampling of DMRs. It might be worthwhile to explore the development of multiplex methylation-specific PCR targeting multiple DMRs as an alternative to a sequencing-based approach. An example outside of the EBV field is the use of methylated *SEPT9* as a blood-based marker for detection of colorectal cancer, with a reported sensitivity of 44.7% for stage I–II diseases in a prospective evaluation^28^. Assay sensitivity is particularly important in the screening setting when low concentrations of circulating tumour DNA and therefore relatively weak cancer signals would be expected for early stage cancers. In the present work, the NPC-associated DMRs were mined from the NPC and the infectious mononucleosis cases described in Fig. 1. This DMR set could be further refined in future studies involving a larger number of cases.

We have previously identified the differentiating quantitative and size profiles of plasma EBV DNA between NPC and non-NPC subjects through non-bisulfite sequencing^17^. The combined analysis of the quantitative and size profiles through non-bisulfite sequencing was shown to allow identification of NPC patients in the screening cohort at an improved specificity of 99.3% and PPV of 19.6%^17^. From the bisulfite sequencing dataset in our current study, the differences in the quantitative and size profiles of plasma EBV DNA between NPC patients and non-NPC subjects could still be observed. Indeed, by just analysing the quantitative and size profiles in the current bisulfite sequencing dataset, we obtained a modelled specificity of 99.1% and PPV of 16.6% in NPC identification. These values were very similar to those reported from the non-bisulfite sequencing analysis. Importantly, this bisulfite sequencing-based technology allowed the combined methylation-, count-, and size-based analysis of bisulfite-treated plasma DNA samples, which offered synergistic effect in the diagnostic performance and boosted the PPV. The prospectively collected plasma samples from our previous screening cohort provided a valuable and unique sample set to test the combined approach. This combined approach was shown to achieve a PPV of 35.1% without compromising the detection sensitivity. These results represent a significant improvement when compared to the reported PPV of 11.0% from the published two time-point PCR-based testing protocol^13^. The substantial improvement in PPV would be translated into a reduction in medical costs on confirmatory investigations. The impact would be pronounced if a mass screening programme based on this combined analysis of plasma EBV DNA is to be launched in endemic regions.

We hypothesised that non-NPC subjects harbour EBV DNA in plasma as a result of viral replication from transient immune suppression^29^. In our previous screening cohort, we observed that there was a correlation between the proportion of screened subjects with plasma EBV DNA positivity, but who did not have NPC, with increasing age^29^. Interestingly, there was also a negative correlation between the proportion of screened subjects with plasma EBV DNA positivity, but who did not have NPC, with the ambient temperature on the day of blood sampling^29^. These results^29^ suggested a possible correlation with weakened immunity which might lead to viral reactivation and replication. Our current study provides another piece of evidence by predicting the origin of viral fragments based on the methylation signatures. We have shown that the plasma EBV DNA molecules from non-NPC subjects were more hypomethylated than those from NPC patients. This molecular feature is in accordance with our understanding that EBV DNA from lytic replication by the viral DNA polymerase is unmethylated^19^. Our current study hence provides epigenetic evidence that the source of EBV DNA in plasma of non-NPC subjects is from viral reactivation.

This work has provided a framework for studying the methylation profiles of circulating viral nucleic acids. We illustrated the potential clinical utility of analysing the plasma EBV DNA methylome in the context of NPC screening. It would be worthwhile to develop EBV DNA methylation markers for other EBV-associated diseases. One potential application may include monitoring of the treatment response to the specific chemotherapy of demethylating agents, e.g., azacytidine, in EBV-associated malignancies^30^. It would also be exciting to extend the concept beyond EBV for studying the methylation profiles of circulating nucleic acids of other oncogenic viruses, for example, hepatitis B virus and human papilloma virus^31^.

In our current study, the total number of NPC patients analysed in both the exploratory and validation cohorts is relatively small, as limited by the number of NPC samples (i.e., 34) from the prospective screening cohort. Therefore, we have included 14 additional NPC samples from an external cohort in the validation set. We thus believe that the robustness of the methylation-based analysis of plasma EBV DNA in differentiating NPC from non-NPC samples is well supported.

In our analysis, within the screening cohort, NPC patients were found to have a lower median total plasma DNA concentration than the non-NPC subjects with transiently or persistently positive plasma EBV DNA results. While it is a belief by some investigators that the concentration of plasma DNA is higher in patients with cancers^32^, this may be more typical for patients with advanced-stage cancers^33–35^. In contrast to this belief, we observed lower concentrations of total plasma DNA in the screening cohort of NPC cases. For this screening cohort, the majority of the NPC cases were early-stage diseases. We also note that the total plasma DNA concentrations were higher in the external cohort with advanced-stage NPC. In the published literature, there is a lack of information on the spectrum of total plasma DNA concentrations in NPC patients. In future studies we may explore the total plasma DNA concentration among NPC patients of different stages in particular those with early stage disease to see if similar findings will be observed. To address the pre-analytical difference in the total plasma DNA concentration as a potential confounder in our study, we have performed the down-sampling analysis of the sequencing data of all the NPC and non-NPC cases in the validation cohort (Supplementary Fig. 5). We have shown similar diagnostic performances of the methylation-based analysis based on the down-sampled number of plasma DNA fragments and that based on all fragments.

In summary, we identified distinct methylation profiles of plasma EBV DNA among different EBV-associated diseases using a viral genome-wide approach. Based on these observations, we analysed the plasma EBV DNA methylome of NPC and non-NPC subjects from a screening cohort. Analysing the differentially methylated EBV DNA sequences allowed differentiation of NPC patients from non-NPC subjects. Together with the other differentiating molecular characteristics, i.e., quantitation and size, the combined approach of plasma EBV DNA analysis demonstrated an enhanced diagnostic performance in NPC identification. Our work has also opened up future clinical applications of genome-wide methylation profiling of plasma EBV DNA in other EBV-associated malignancies, and indeed for other viral-associated cancers.

## Methods

### Clinical samples

The study was approved by the Joint Chinese University of Hong Kong—Hospital Authority New Territories East Cluster Clinical Research Ethics Committee.

In the clustering analysis, we included 15 patients with NPC, 9 patients with EBV-associated lymphoma and 5 patients with infectious mononucleosis. All cancer patients presented symptomatically and were recruited from the Department of Clinical Oncology and the 5 patients with infectious mononucleosis were recruited from the Department of Otorhinolaryngology, Head and Neck Surgery of Prince of Wales Hospital in Hong Kong. All of them consented for sequencing analysis of their plasma samples. The EBV status for the tumours was confirmed by in situ hybridisation for EBV-encoded small RNAs on tumour tissues.

Archived plasma samples of patients with NPC and non-NPC subjects from the published prospective screening cohort were used for methylation analysis in the current study.

The protocol of the screening study has been described in detail before^13^. We recruited subjects who were ethnically Chinese males aged between 40 and 62 years and did not show symptoms of NPC. We excluded subjects with history of cancer or autoimmune diseases and those who were receiving systemic glucocorticoids or immunosuppressive therapy. We adopted a two time-point testing protocol of plasma EBV DNA detection by the same real-time PCR assay, which targeted the *Bam*HI-W region of the EBV genome. In the two time-point testing protocol, subjects who were positive for plasma EBV DNA at baseline would be retested by the same PCR assay. Subjects who were also positive at a follow-up test would be considered as screen-positive and referred for confirmatory investigations. A venous blood sample of 20 mL was collected from each participant at enrolment. Totally, 800 μL of plasma were used for the PCR-based analysis. The remaining plasma samples were stored at −80 ^º^C. All the participants provided written informed consent for sequencing analysis of the plasma samples.

The clinical staging of NPC patients was based on the MRI findings according to the tumour-node-metastasis cancer staging system of the American Joint Committee on Cancer (AJCC) (seventh edition). The clinical staging of lymphoma patients was based on the Ann-Arbor staging system.

### Study design

The study design is described with reference to the REMARK guidelines^36^. The exploratory and validation cohorts consisted of samples of NPC and non-NPC subjects randomly selected from the prospective screening study (simple randomisation). The exploratory cohort consisted of 10 NPC patients and 40 non-NPC subjects (20 with transiently positive and 20 with persistently positive plasma EBV DNA results) randomly selected from the screening study. The validation cohort consisted of the remaining 23 NPC patients and 120 randomly selected non-NPC subjects (90 with transiently positive and 30 with persistently positive plasma EBV DNA results). There is no significant difference in the mean plasma EBV DNA concentrations measured by the real-time PCR analysis between the 120 non-NPC subjects in the validation set and all the 1078 non-NPC subjects from the screening cohort (Student’s *t* test, *p* = 0.4). There is no statistically significant difference in the stage distribution (stages I and II versus stages III and IV) of NPC patients from the screening cohort between the exploratory and validation sets (Fisher’s exact test, *p* = 1.0). Samples in the two cohorts did not overlap. The validation analysis was performed in a blinded manner. In the training phase, we first performed isolated methylation-, count- and size-based analyses of the bisulfite sequencing data of NPC and non-NPC samples from the exploratory cohort. The definitions of the methylation-, count- and size-based parameters in the corresponding analyses were locked down before validation. Cutoff values of these three parameters were determined solely based on the exploratory dataset with reference to the means and standard deviations and were also locked down before validation. Then, we performed the combined methylation-, count- and size-based analysis in the exploratory dataset. In the combined analysis, a plasma sample was flagged as positive if its sequencing data concurrently passed the three corresponding cutoffs. The analytical definitions of the combined analysis and the cutoffs were locked down before validation. The diagnostic performances (sensitivity and specificity) of the isolated and combined analyses were subsequently evaluated in the validation cohort.

### Power analysis

A power analysis was performed to determine the number of non-NPC subjects required in the validation sample set given that we analysed 23 NPC patients from the screening cohort. Based on the means and standard deviations of the EBV DNA methylation scores of NPC patients and non-NPC subjects in the exploratory sample set, if we would reproduce the observed difference in the validation set at a significant level of 0.001 and a power of 0.999, at least 82 non-NPC subjects would be required.

### Blood sample collection and plasma DNA extraction

Peripheral blood samples were collected into EDTA tubes and immediately stored at 4 °C before further processing. The centrifugation protocol was as follow: centrifugation at 1600*g* for 10 min at 4 °C then re-centrifugation of the plasma portion at 16,000*g* for 10 min at 4 °C to remove the residual blood cells. All the plasma samples were stored at −80 °C. For all the samples analysed, plasma DNA was extracted from 4 mL of plasma. DNA from plasma was extracted using the QIAamp Circulating Nucleic Acid Kit (Qiagen).

### DNA library construction and bisulfite conversion

Plasma DNA libraries were constructed using the TruSeq Nano DNA Library Preparation Kit (Illumina) according to the manufacturer’s protocol. The adaptor-ligated DNA molecules were subjected to two rounds of bisulfite treatment using Epitect Plus Bisulfite Kit (Qiagen). The protocol of two-round bisulfite treatment^37–41^ was shown to achieve a high conversion rate with minimal intra- and inter-individual variabilities. After bisulfite treatment, the DNA was amplified with 13 cycles of PCR using the KAPA HiFi Uracil + ReadyMix PCR Kit (KAPA Biosystems).

### Enrichment of EBV DNA molecules

Target enrichment of EBV DNA molecules from the plasma DNA samples was done via the capture-probe system. We designed the EBV capture probes to cover the entire EBV genome, which were then synthesised by Roche NimbleGen (SeqCap EZ Developer, Roche NimbleGen Inc). The probes would be specifically designed to capture both converted (unmethylated) and unconverted (methylated) DNA fragments after bisulfite treatment. Multiplexing of DNA libraries from four plasma samples in one capture reaction were performed. Equal amounts of DNA libraries for each sample were used. We had also included probes to cover human autosomal regions from all, except the sex chromosomes, as a reference set. The BED file with the EBV and autosomal regions for the capture-probe design is included in the Supplementary Information (Supplementary Data 2). A probe mixture containing the molar ratio of EBV probes to autosomal DNA probes in the ratio of 100:1 was used in each capture reaction because the viral DNA molecules were expected to be a minority proportion of the DNA in the samples. The commercial vendor provided us the EBV and autosomal probe mixtures in solution form according to our requested ratio. The captured DNA libraries were re-amplified with 14 cycles of PCR.

Two plasma DNA samples (TBR1765 and TBR2003) from two male patients with advanced NPC were evaluated with serial 10-fold dilutions (1:1, 1:10, 1:100, 1:1000 and 1:10,000). The measured concentrations of EBV DNA by quantitative PCR for samples TBR1765 and TBR2003 were 24,625 and 26,750 copies/mL, respectively. The diluent for the serial dilution consisted of plasma DNA from a separate healthy male individual for each of the NPC sample. The concentrations of plasma DNA from the two healthy control plasma samples for dilutions of TBR1765 and TBR2003 were 955 and 1182 copies/mL, respectively. A linear relationship between the number of sequenced EBV DNA fragments and the dilution factor could be observed for the range of 500–1.5 million EBV DNA fragments (Supplementary Fig. 6). We observed consistent values of EBV DNA methylation scores across the whole range of dilutions for both samples (Supplementary Table 3).

### Sequencing of DNA libraries

The multiplexed DNA libraries were sequenced using the NextSeq 500 system (Illumina). A 76 × 2 paired-end sequencing protocol was used.

### Bioinformatics analysis

The paired-end bisulfite sequencing data were analysed by the Methy-Pipe^42^ developed by our group to perform sequence alignment and methylation call. The sequence reads were aligned to the combined reference genomes including reference human genome (hg19) and EBV genome (AJ507799.2). The methylation densities of all the CpG sites across the viral genome were deduced. The DMRs were mined based on the criteria described in the main text. The quality control statistics for all the cases in Fig. 1 and the exploratory and validation cohorts were described in Supplementary Data 3.

### Mining of NPC-associated DMRs

For the identification of NPC-associated DMRs, we defined a CpG site as differentially methylated if the methylation density of the site was >80% in the pooled sequencing data of 15 NPC cases and <60% in the pooled data of 5 infectious mononucleosis cases shown in Fig. 1. Such cutoffs were chosen based on the performances of methylation-based analysis in the exploratory sample set using different DMR sets derived from different cutoffs. We derived the EBV DNA methylation scores of all the NPC and non-NPC samples in the exploratory set using the different sets of DMRs. Using the strategy of defining a cutoff value in the EBV DNA methylation scores (i.e., 3 standard deviations below the mean), we evaluated the performances of methylation-based analysis in the exploratory sample set with different DMR sets. The sensitivities of the methylation-based analysis based on all DMR sets derived from the different cutoffs were 100%. The specificities of the methylation-based analysis with the different DMR sets at the corresponding methylation density cutoffs are shown in Supplementary Fig. 7. As shown in the figure, the specificity of the methylation-based analysis is highest when the cutoff of methylation density is set at 80% for NPC and at 60% or 40% for IM, or at 70% for NPC and at 60% for IM, or at 50% for NPC and at 30% for IM, or at 40% for NPC and at 30% for IM, using the cutoff defined at 3 standard deviations from the mean. The result suggests that these DMR sets have the highest differentiating power and we decided to choose the DMR set defined with the methylation density of NPC being greater than 80% and that of IM being less than 60%.

### Calculation of EBV DNA methylation score

All the sequenced EBV DNA reads within DMRs would be used to determine the total depths at CpG dinucleotides within the DMRs. The fraction of unconverted cytosines (methylated) over the sum of unconverted cytosines (methylated) and converted thymine (unmethylated) present in its total depth at CpG dinucleotides within DMRs was used to define the methylation score as shown by Eq. (1). If one EBV DNA read spans across multiple CpG sites, only those CpG sites within a DMR would be included for the calculation (i.e., at the level of genomic regions). The score was derived using a non-weighted approach (i.e., equal weights to all the CpG sites within the 158 DMRs). Supplementary Fig. 1 illustrates the calculation of the EBV DNA methylation score (Eq. (1)).

### Defining the cutoffs for the analyses

Cutoff values of the three parameters in the methylation-, count- and size-based analyses were determined solely based on the exploratory dataset with reference to the means and standard deviations. In the methylation-based analysis, a cutoff value in the EBV DNA methylation score was defined at 3 standard deviations below the mean of the methylation scores of these 10 NPC patients in the exploratory dataset. In the count-based analysis, a cutoff value was defined at 3 standard deviations below the mean of the logarithmic values of  the portion of EBV DNA fragments of the same 10 NPC patients, assuming EBV DNA quantity follows the Gaussian mixture distribution. In the size-based analysis, a cutoff value was defined at 3 standard deviations above the mean values of the EBV size ratios of all the 10 NPC patients.

### DMR down-sampling analysis

We performed a DMR down-sampling analysis with 100 simulations. In the analysis, with the serial down-sampling of the 158 DMRs, we evaluated the AUROC values of the methylation-based analysis at the corresponding number of DMRs in the validation cohort using the statistical R package pROC. As shown in the Supplementary Fig. 3, we could observe a decline in the AUROC values in NPC detection with serial down-sampling of DMRs.

### Plasma DNA down-sampling analysis in the validation cohort

Noting the different amounts of plasma DNA input for different cases, we performed a down-sampling analysis of the sequencing data for the NPC and non-NPC cases in the validation cohort to evaluate the potential impact of sequencing depth on the performance of the methylation-based analysis. From the raw sequencing data, we first removed PCR duplicates of total mapped DNA fragments of all NPC and non-NPC samples in the validation cohort to obtain what would subsequently be termed deduped fragments. The total number of deduped fragments obtainable for 95% of the cases (NPC and non-NPC) in the validation cohort was 9,383,179. We then down-sampled the deduped fragments from all cases to 9,383,179 reads per case. Seven cases in the validation cohort had deduped fragment counts lower than this level and were excluded from the down-sampling analysis. Then we calculated the EBV DNA methylation score on the down-sampled reads for each included case. As shown in the Supplementary Fig. 5, we could still observe that NPC patients from both the screening cohort (median = 86.4, IQR: 82.9–87.3) and the external cohort (median = 87.6, IQR: 86.5–88.0) had significantly higher EBV DNA methylation scores than non-NPC subjects with transiently positive (median = 66.8, IQR: 59.3–71.3) and persistently positive (median = 68.7, IQR: 62.2–71.1) results in this down-sampling analysis (Kruskal–Wallis test, *p* < 0.0001). The performance of the methylation-based analysis based on the down-sampled reads (sensitivity: 100%, specificity: 82.6%) is comparable to that based on all fragments (sensitivity: 100%, specificity: 83.3%).

We have also performed down-sampling of EBV DNA fragments among NPC and non-NPC samples in the validation set. Plasma EBV DNA fragments were down-sampled to equal levels (50, 100, 300, 500, 700 and 900 EBV DNA fragments) to ensure similar viral genome coverage for different samples. The EBV DNA methylation scores were then calculated using the same down-sampled amount of EBV DNA fragments in each scenario (Supplementary Fig. 8). At different levels of down-sampled EBV DNA fragments, the methylation-based analysis based on the EBV DNA methylation score could still differentiate NPC from non-NPC samples, while higher EBV DNA methylation scores were observed among NPC samples from both the screening and external cohorts compared to non-NPC samples. In addition, we have evaluated the diagnostic performances of isolated count-based and combined count- and methylation-based analyses in the validation cohort using AUROC analysis (Supplementary Fig. 9). The combined count- and methylation-based analysis (0.988) achieved a significantly higher AUROC value than the isolated count-based analysis (0.938) (Bootstrap test, *p* < 0.001). These results suggested that the methylation-based analysis could provide additional differentiating power of NPC from non-NPC samples independent of count-based analysis.

### Statistical analysis

The Kruskal–Wallis test was used to compare the EBV DNA methylation scores, proportion of EBV DNA reads and EBV DNA size ratios in the three groups. A *p* value of <0.05 was considered as statistically significant.

### Reporting summary

Further information on research design is available in the Nature Research Reporting Summary linked to this article.

## Supplementary information

Supplementary Information

Supplementary Data 1

Supplementary Data 2

Supplementary Data 3

Reporting Summary

Description of Additional Supplementary Files

Source Data

## Data Availability

Sequence data for all the subjects studied in this work have been deposited in the European Genome-Phenome Archive (EGA) with the accession code EGAS00001003408. The source data underlying Figs. 3 and 4 are provided as a Source Data file.
