# Supplementary material for: Methylation analysis of plasma DNA informs etiologies of Epstein-Barr virus-associated diseases
Source: Nat Commun. 2019 Jul 22;10:3256. doi: 10.1038/s41467-019-11226-5 (PMC6646310; doi:10.1038/s41467-019-11226-5)
Supplement: Supplementary file 1 — Supplementary Information [file 41467_2019_11226_MOESM1_ESM.pdf]

Supplementary Information

Methylation analysis of plasma DNA informs etiologies of Epstein-Barr virus-associated diseases

Authors: W.K. Jacky Lam, Peiyong Jiang, K.C. Allen Chan, Wenlei Peng, Huimin Shang, Macy M.S. Heung, Suk Hang Cheng, Haiqiang Zhang, O.Y. Olivia Tse, Radha Raghupathy, Brigette B.Y. Ma, Edwin P. Hui, Anthony T.C. Chan, John K.S. Woo, Rossa W.K. Chiu, Y.M. Dennis Lo

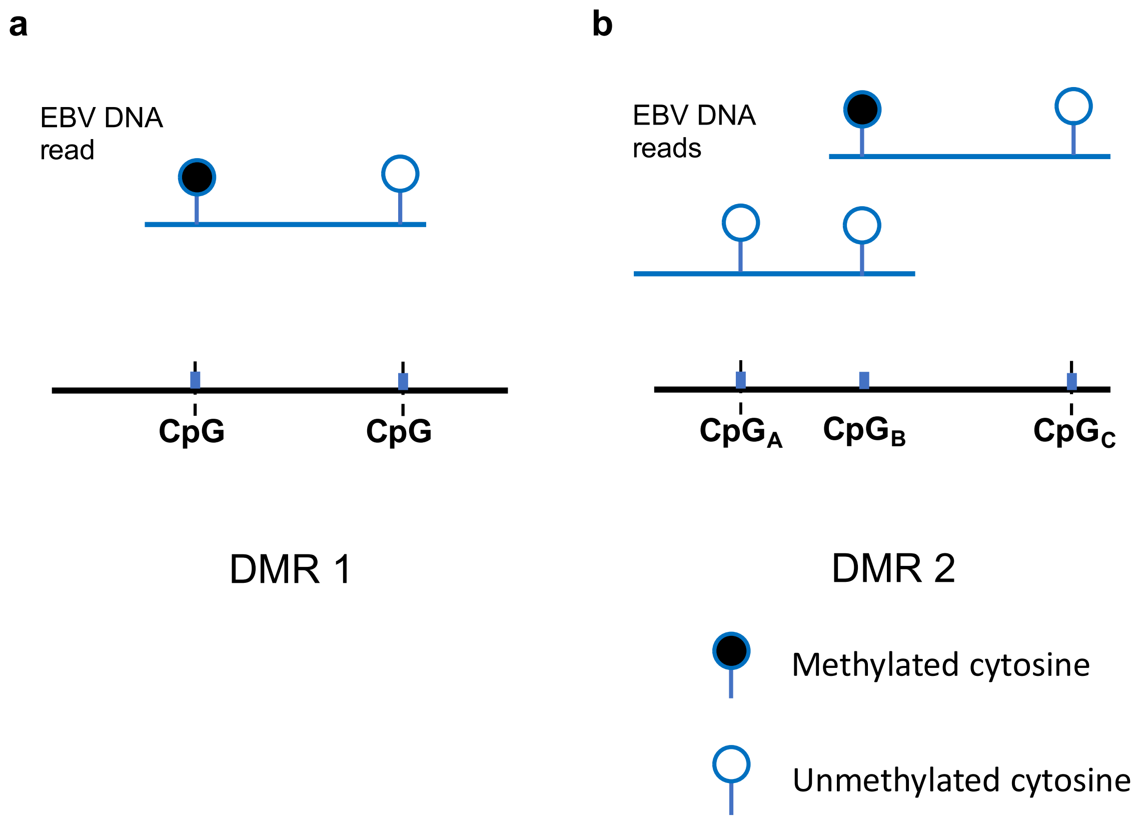

Supplementary Fig. 1 An illustration for the calculation of EBV DNA methylation score. **a** A hypothetical DMR 1 consists of 2 CpG sites and there is only one EBV DNA read which covers the DMR 1. This EBV DNA read has one methylated cytosine and one unmethylated cytosine. The EBV DNA methylation score for DMR 1 would be 1 (methylated) / 1 (methylated) + 1 (unmethylated) x 100 = 50. **b** A hypothetical DMR 2 consists of 3 CpG sites and there are two EBV DNA reads which cover DMR 2. There is one unmethylated cytosine on CpG_A, one methylated and one unmethylated cytosines on CpG_B, one unmethylated cytosine on CpG_C. The EBV DNA methylation score for DMR 2 would be 1 (methylated) / 1 (methylated) + 3 (unmethylated) x 100 = 25. The aggregated EBV DNA methylation score for DMR 1 and 2 would be 2 (methylated) / 2 (methylated) + 4 (unmethylated) x 100 = 33.3.

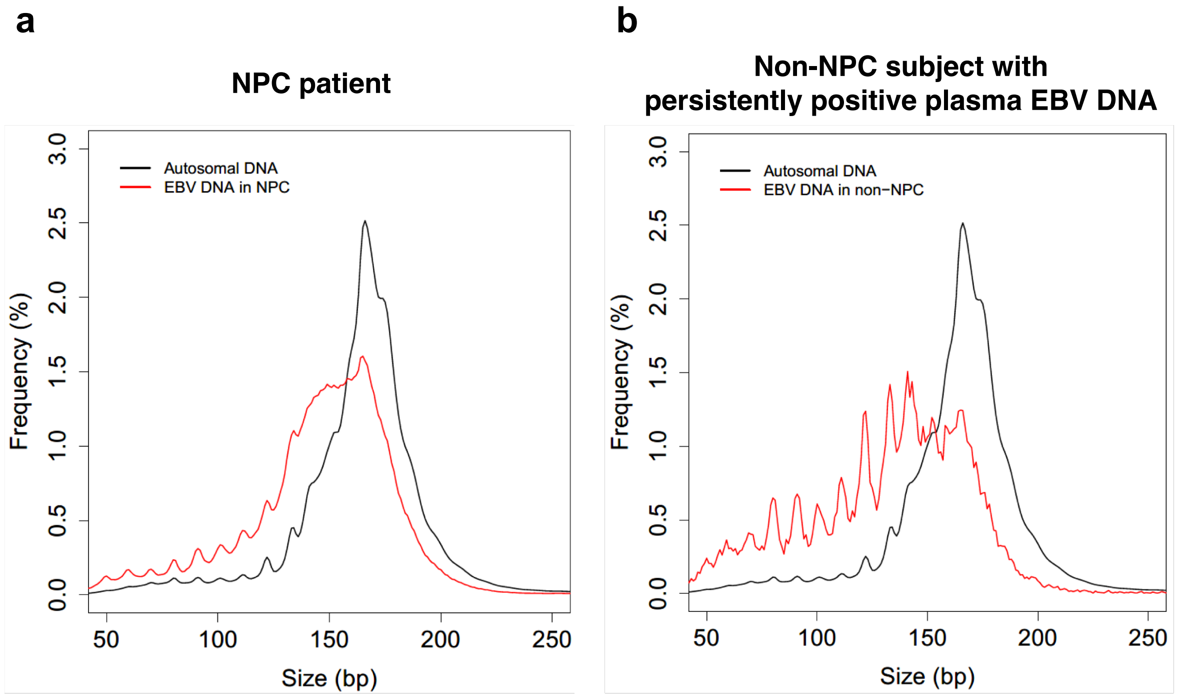

Supplementary Fig. 2 Size distributions of EBV DNA (red curve) and autosomal DNA (black curve) in the plasma of **a** a patient with NPC and **b** a non-NPC subject with persistently positive plasma EBV DNA results.

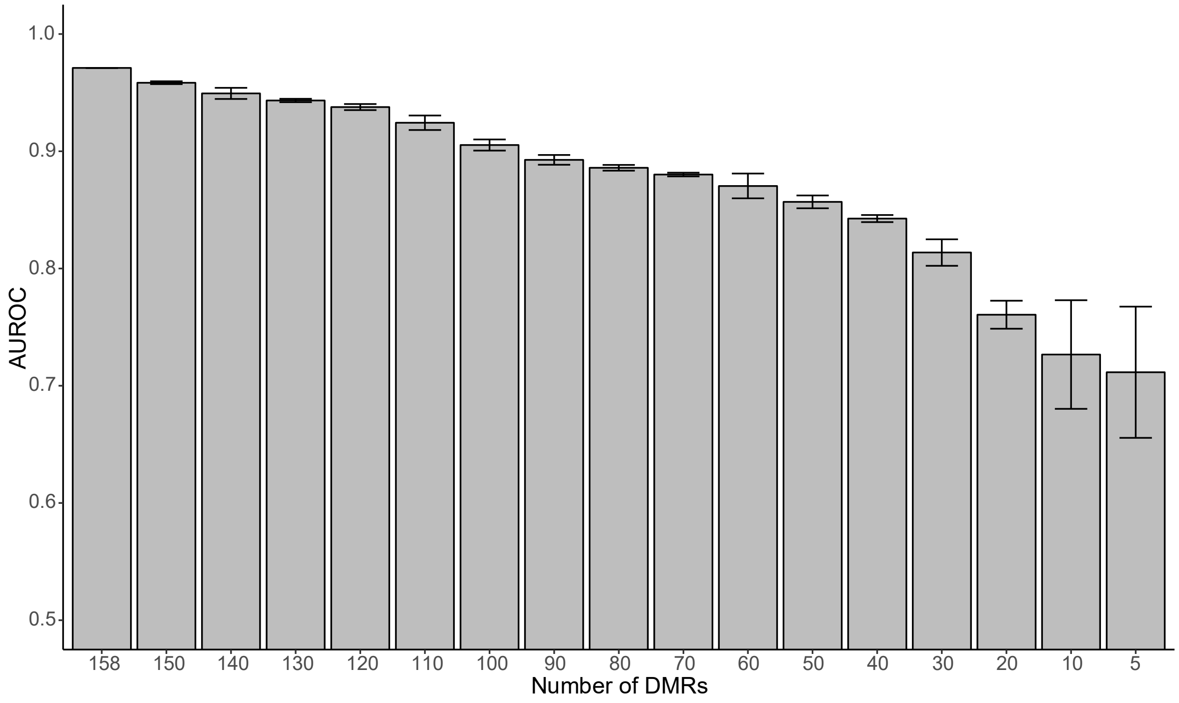

Supplementary Fig. 3 Differentially methylated regions (DMRs) down-sampling analysis. Area under the receiver operating characteristic (AUROC) values of methylation-based analysis in the DMRs down-sampling analysis are shown. The error bar indicates the standard deviation.

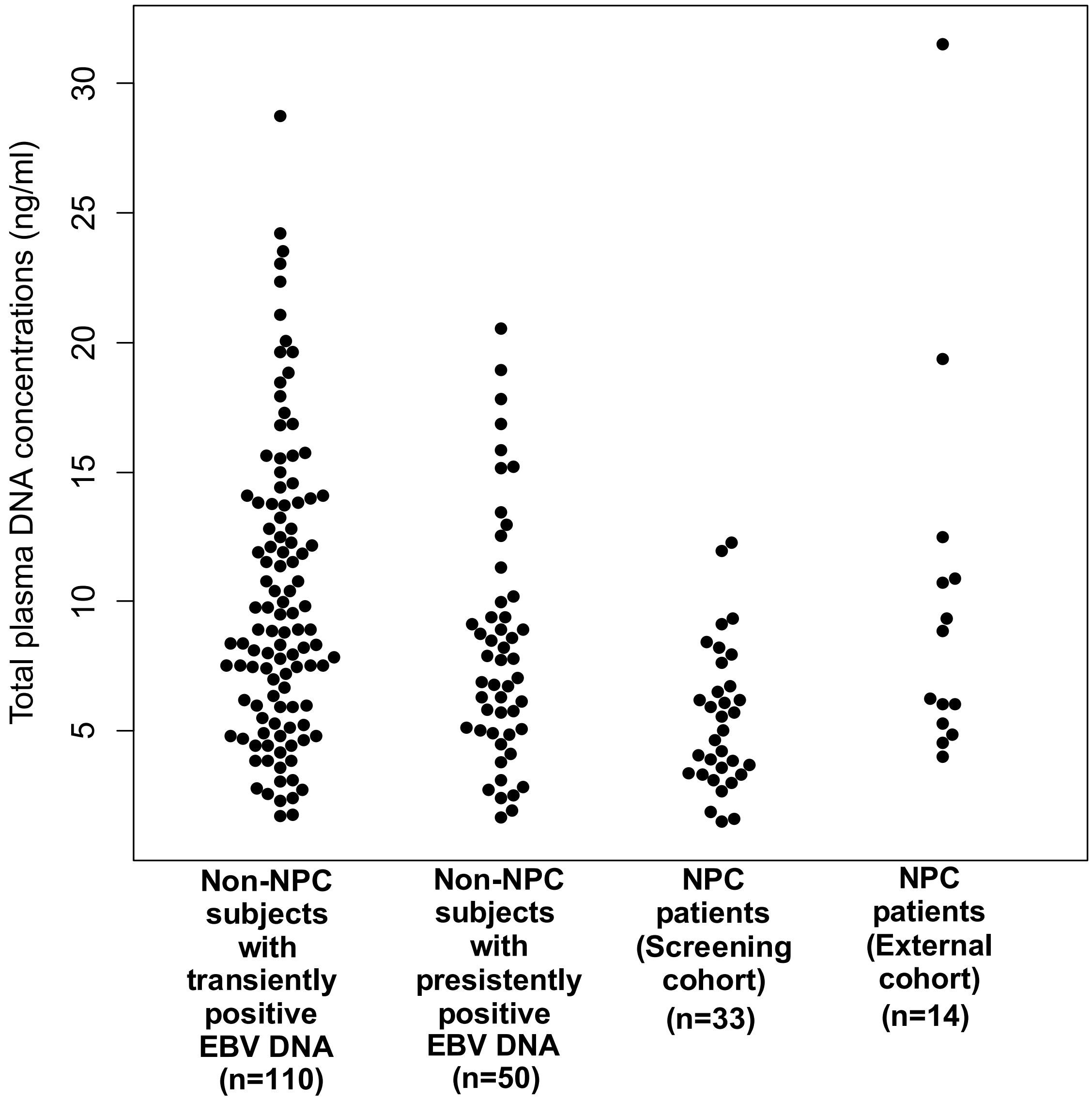

Supplementary Fig. 4 Total plasma DNA concentrations for the NPC and non-NPC samples in the screening and external cohorts.

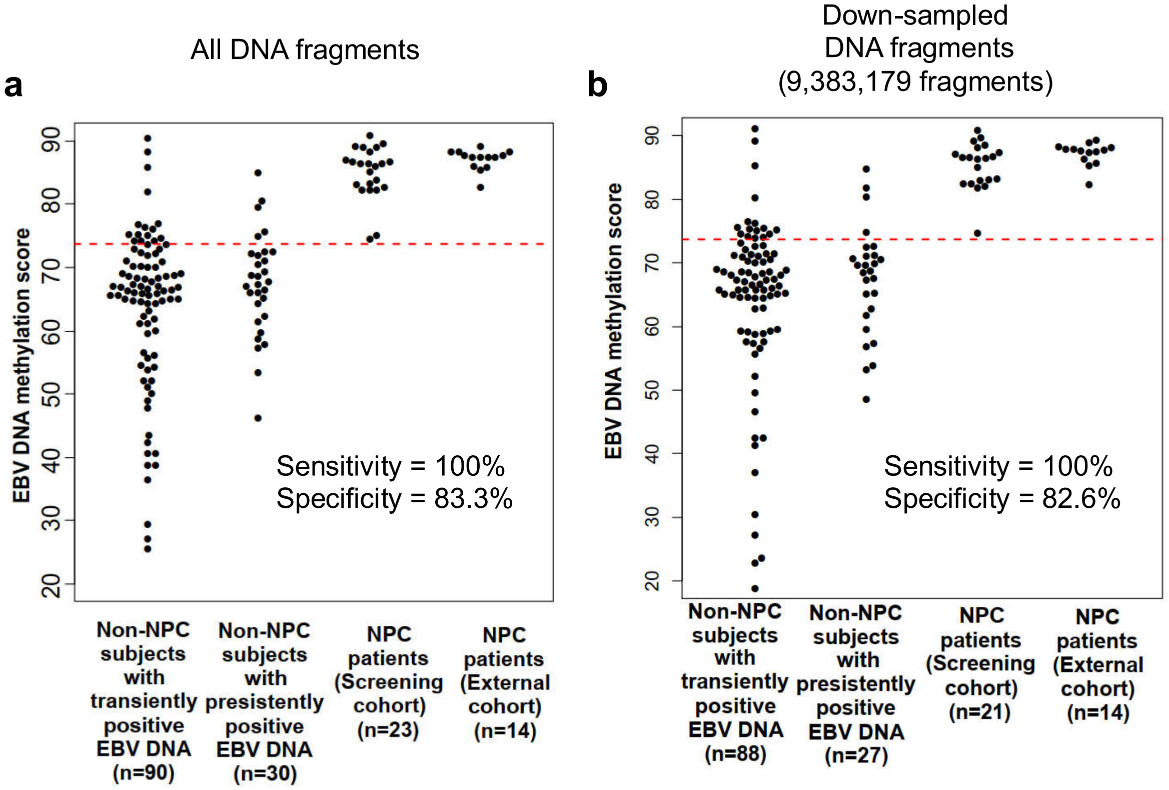

Supplementary Fig. 5 Plasma DNA down-sampling analysis. Methylation-based analysis of plasma EBV DNA in the validation sample set based on all sequenced fragments (autosomal and viral) and down-sampled level of deduped fragments are shown. The cutoff value in the EBV DNA methylation score defined in the exploratory dataset is denoted by the red dotted line. **a** The EBV DNA methylation scores of the NPC patients (from both the screening and external cohorts) and non-NPC subjects with transiently positive and persistently positive results are calculated based on all sequenced fragments. **b** The EBV DNA methylation scores of the same group of NPC patients (from both the screening and external cohorts) and non-NPC subjects with transiently positive and persistently positive results are calculated based on the down-sampled level of deduped fragments (i.e. 9,383,179 fragments).

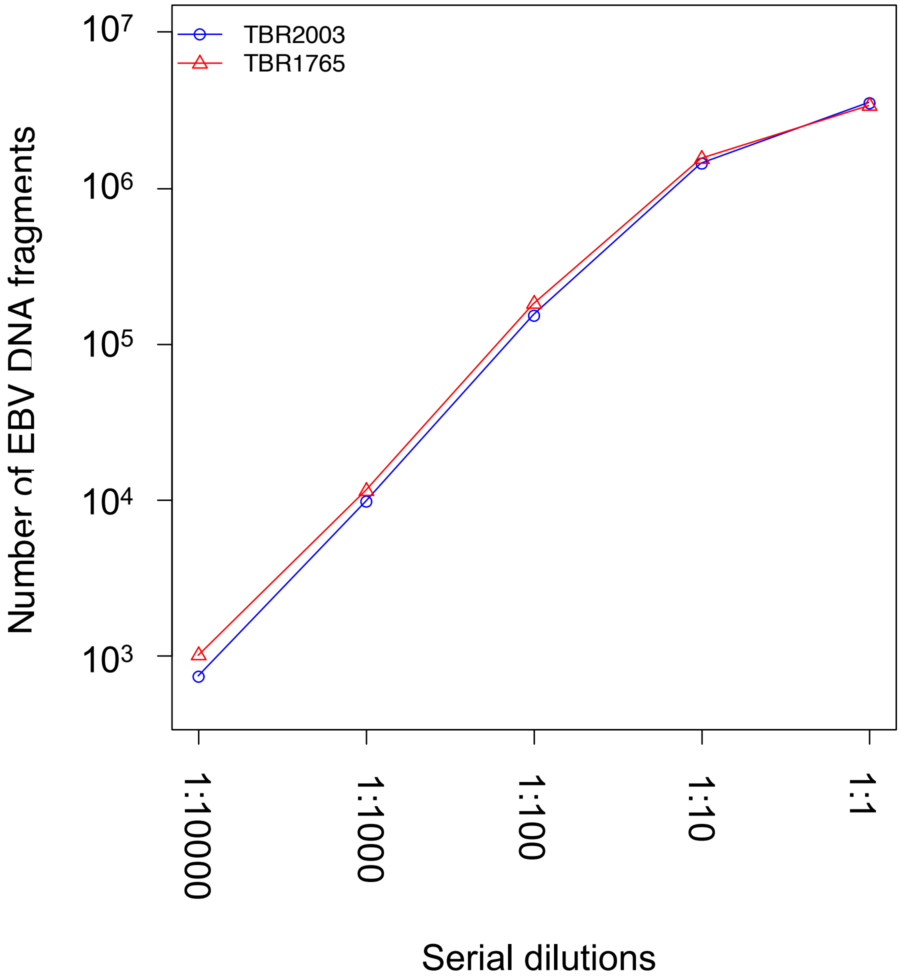

Supplementary Fig. 6 Serial dilution experiment. Number of EBV DNA fragments recovered by targeted sequencing with the capture probes panel in the serial dilution experiment of 2 plasma samples from 2 patients with advanced NPC (TBR1765 and TBR2003). Serial 10-fold dilutions (1:1, 1:10, 1:100, 1:1,000 and 1:10,000) of the 2 plasma DNA samples were performed. A linear relationship between the number of sequenced EBV DNA fragments and the dilution factor could be observed for the range of 500 to 1.5 million EBV DNA fragments.

	NPC>80%	NPC>70%	NPC>60%	NPC>50%	NPC>40%	NPC>30%	NPC>20%	NPC>10%
IM<10%	0.175	0.25	0.35	0.325	0.275	0.175	0.175	0.375
IM<20%	0.225	0.45	0.475	0.475	0.475	0.45	0.4	
IM<30%	0.725	0.75	0.675	0.775	0.775	0.75		
IM<40%	0.775	0.75	0.7	0.725	0.7			
IM<50%	0.75	0.725	0.75	0.7				
IM<60%	0.775	0.775	0.75					
IM<70%	0.675	0.675						
IM<80%	0.625							

Supplementary Fig. 7 Determination of methylation density cutoffs criteria in the NPC and IM cases for mining DMRs. Heatmap showing the specificities of the methylation-based analysis in the exploratory sample set with the different DMR sets at the corresponding methylation density cutoffs. Each box represents the DMR set derived from the different cutoffs in the 15 NPC and 5 IM cases. In the methylation-based analysis, the EBV DNA methylation scores of all the NPC and non-NPC samples in the exploratory set were derived using the different sets of DMRs. Using the strategy of defining a cutoff value in the EBV DNA methylation scores (i.e. 3 standard deviations (SD) below the mean), the performances of methylation-based analysis in the exploratory sample set with different DMR sets were evaluated. The number in the box refers to the specificity of methylation-based analysis based on the corresponding DMR sets. The sensitivities of the methylation-based analysis based on all DMR sets listed were 100%. (IM = infectious mononucleosis)

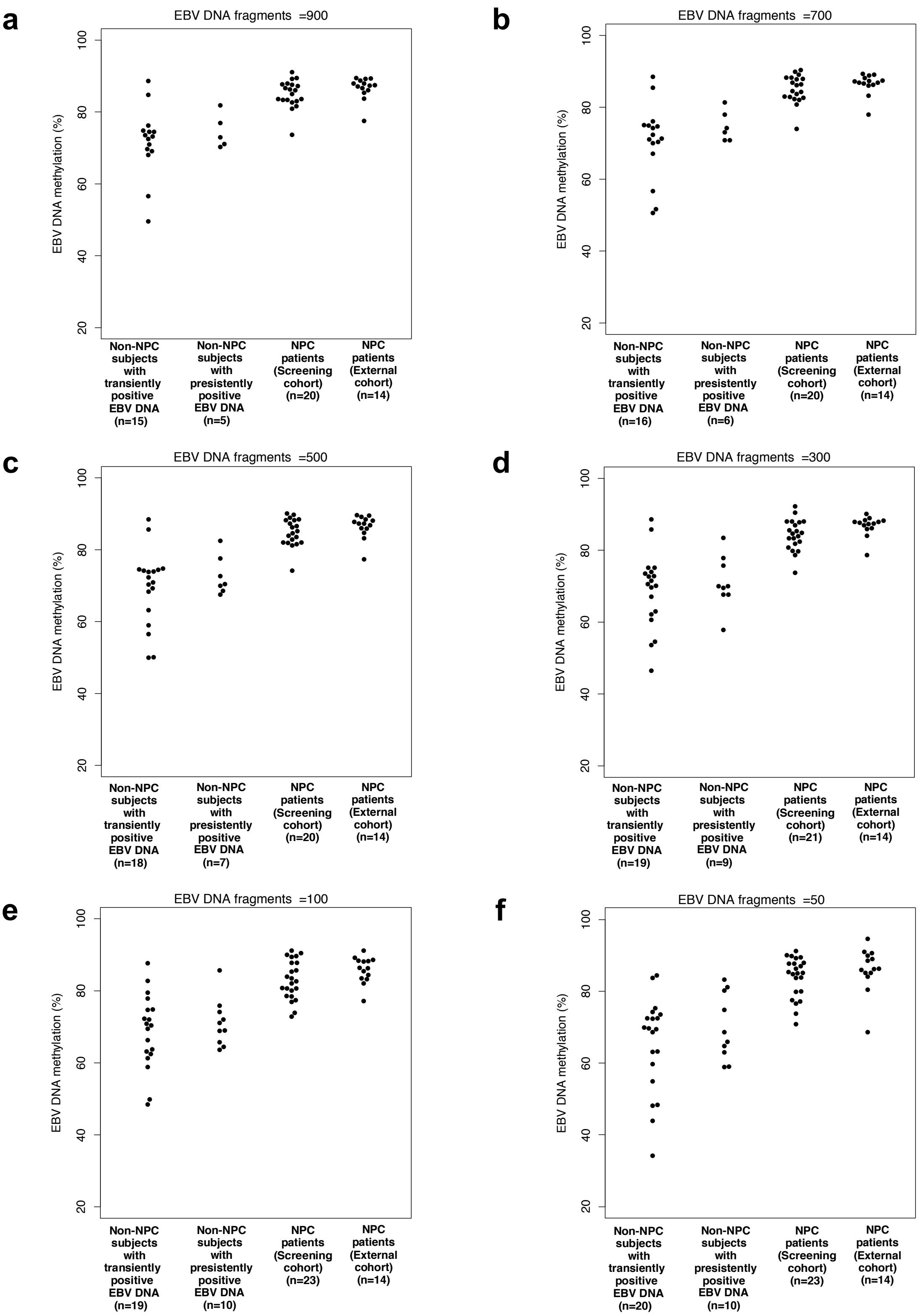

Supplementary Fig. 8 Plasma EBV DNA down-sampling analysis. Methylation-based analysis of plasma EBV DNA in the validation sample set based on down-sampled EBV

DNA fragments are shown. The EBV DNA methylation scores of the NPC patients (from both the screening and external cohorts) and non-NPC subjects with transiently positive and persistently positive results are calculated based on different levels of down-sampled EBV DNA fragments. Samples with EBV DNA fragments less than the down-sampled level were excluded. EBV DNA fragments were down-sampled to **a** 900 fragments, **b** 700 fragments, **c** 500 fragments, **d** 300 fragments, **e** 100 fragments and **f** 50 fragments.

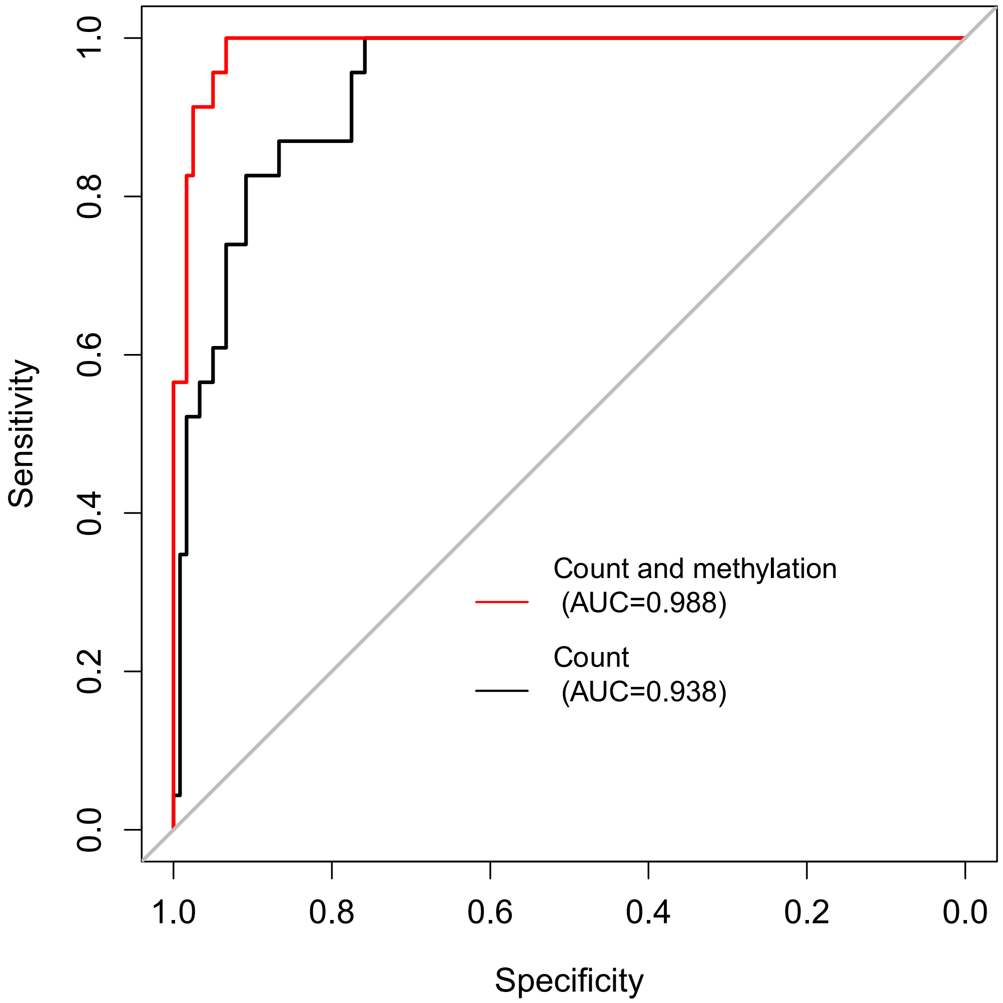

Supplementary Fig. 9 Diagnostic performances of the isolated and combined analyses. Receiver operating characteristics (ROC) curve analysis of the isolated count-based and the combined methylation- and count-based analyses are shown. Area under the curve (AUC) values are shown. The combined analysis involving the methylation- and count-based parameters achieved a significantly higher AUC value than the combined analysis involving the count- and size-based analyses only (Bootstrap test, $p<0.001$).

	NPC	EBV-associated lymphoma	Infectious mononucleosis
**Number**	15	9	5
**Sex**			
**M**	11	7	1
**F**	4	2	4
**Median age, year (Range)**	53<br>(45– 61.5)	55<br>(24 – 67)	17<br>(7 – 23)
**Tumour stage**			
**I**	2	3	NA
**II**	0	0	NA
**III**	6	0	NA
**IV**	7	6	NA

Supplementary Table 1 Characteristics of patients with different EBV-associated diseases. (NA = not applicable)

DMR	Start	End	Remarks
1	10010	10045	
2	100497	102655	
3	102898	103837	
4	10301	11852	
5	104745	104794	
6	105003	106191	
7	106491	107106	
8	107456	107457	
9	107896	108274	
10	108654	108870	
11	109213	109214	
12	109506	109507	
13	109812	109976	
14	110766	110767	
15	111149	111150	
16	112125	112126	
17	112612	112621	
18	112969	112970	
19	113730	113928	
20	114570	114845	
21	115303	115430	
22	115701	115702	
23	115910	115911	
24	116478	116479	
25	116827	116828	
26	117588	117678	
27	117942	117943	
28	118412	118413	
29	118631	118689	
30	118976	118982	
31	119912	119916	
32	120156	120157	
33	120444	120445	
34	12193	12371	
35	122163	122164	
36	122365	122547	
37	122830	123030	
38	123738	123753	
39	124046	124484	
40	125439	125638	
41	126134	126135	

42	126369	126392	
43	126664	127176	
44	127411	127412	
45	127880	128104	
46	128401	128402	
47	129441	129604	
48	130560	130561	
49	131349	131350	
50	131969	131970	
51	132278	132279	
52	132535	132545	
53	132834	133283	
54	133569	134156	
55	134390	134997	
56	135320	135365	
57	135569	138149	
58	1388	1413	
59	139123	140079	
60	140345	140813	
61	143229	143434	
62	143641	143700	
63	145706	145709	
64	145938	146062	
65	146266	146301	
66	146567	146568	
67	147677	148175	
68	148548	151550	
69	151854	156112	
70	156322	165096	
71	165320	165486	
72	1654	1655	
73	166720	166721	*
74	167779	167929	*
75	168162	169007	*
76	1892	1893	
77	2121	5686	
78	36183	36274	*
79	36518	36657	*
80	36903	36923	*
81	37206	37316	*
82	37567	37568	*
83	37893	38348	

84	39779	40516	
85	40780	40960	
86	41374	41894	*
87	42138	42387	
88	42896	43104	*
89	44022	44023	
90	44268	44269	
91	44548	44639	
92	45232	45233	
93	46020	46066	
94	46357	46358	
95	46697	47536	
96	47796	48281	
97	48547	49890	
98	50637	51222	
99	51434	52942	
100	53209	53602	
101	53814	55006	
102	55342	55861	
103	56323	56584	
104	57192	57193	
105	57951	58334	
106	58593	63903	
107	589	702	
108	6270	6300	
109	64393	64522	
110	65185	65350	
111	65608	65609	
112	66058	66442	
113	66688	66689	
114	66976	66977	
115	67187	67188	
116	68205	68507	
117	68710	68711	
118	69423	69424	
119	70146	70147	
120	70999	71000	
121	71482	71483	
122	71923	71924	
123	72638	72652	
124	72989	72990	
125	73327	74511	

126	75144	75961	
127	76368	76371	
128	77065	77087	
129	77515	77516	
130	78373	78374	
131	80166	80714	
132	80968	81021	
133	81739	81766	
134	82089	82346	
135	83871	84054	*
136	84335	84338	*
137	84770	84771	*
138	84988	85538	*
139	85744	85794	*
140	86887	86904	*
141	87277	87335	*
142	87538	88082	*
143	88303	88502	*
144	89012	89013	*
145	91532	91944	
146	92153	92178	
147	92408	92709	
148	93387	93502	
149	94358	94359	
150	94873	95267	
151	9516	9681	
152	95871	95872	
153	96675	96953	
154	97175	97176	
155	97463	97791	
156	98375	98523	
157	99230	99368	
158	99930	99931	

* = correlate with the latency associated genes based on the genomic location

Supplementary Table 2 Genomic coordinates of the 158 nasopharyngeal carcinoma (NPC)-associated differentially methylated regions (DMRs).

**Sample**	**Dilutions**	**Number of EBV DNA fragments**	**EBV DNA methylation score**
TBR1765	1:1	3372691	87.5
	1:10	1550498	87.9
	1:100	181002	85.1
	1:1000	11531	87.6
	1:10000	1009	84.9
TBR2003	1:1	3530693	87.4
	1:10	1443135	88.0
	1:100	153759	85.2
	1:1000	9785	88.0
	1:10000	744	87.3

Supplementary Table 3 Table showing the number of EBV DNA fragments and EBV DNA methylation scores for the 2 NPC plasma samples (TBR1765 and TBR2003) in the serial dilution experiment.
