# Supplementary material for: Methylation analysis of plasma DNA informs etiologies of Epstein-Barr virus-associated diseases
Source: Nat Commun. 2019 Jul 22;10:3256. doi: 10.1038/s41467-019-11226-5 (PMC6646310; doi:10.1038/s41467-019-11226-5)
Supplement: Supplementary file 5 — Reporting Summary [file 41467_2019_11226_MOESM5_ESM.pdf]

nature research

Corresponding author(s): Y.M. Dennis LO

Last updated by author(s): Jun 28, 2019

Reporting Summary

Nature Research wishes to improve the reproducibility of the work that we publish. This form provides structure for consistency and transparency in reporting. For further information on Nature Research policies, see Authors & Referees and the Editorial Policy Checklist.

Statistics

For all statistical analyses, confirm that the following items are present in the figure legend, table legend, main text, or Methods section.

n/a	Confirmed	
☐	☒	The exact sample size (*n*) for each experimental group/condition, given as a discrete number and unit of measurement
☐	☒	A statement on whether measurements were taken from distinct samples or whether the same sample was measured repeatedly
☐	☒	The statistical test(s) used AND whether they are one- or two-sided<br>*Only common tests should be described solely by name; describe more complex techniques in the Methods section.*
☐	☒	A description of all covariates tested
☐	☒	A description of any assumptions or corrections, such as tests of normality and adjustment for multiple comparisons
☐	☒	A full description of the statistical parameters including central tendency (e.g. means) or other basic estimates (e.g. regression coefficient) AND variation (e.g. standard deviation) or associated estimates of uncertainty (e.g. confidence intervals)
☐	☒	For null hypothesis testing, the test statistic (e.g. *F*, *t*, *r*) with confidence intervals, effect sizes, degrees of freedom and *P* value noted<br>*Give P values as exact values whenever suitable.*
☒	☐	For Bayesian analysis, information on the choice of priors and Markov chain Monte Carlo settings
☒	☐	For hierarchical and complex designs, identification of the appropriate level for tests and full reporting of outcomes
☒	☐	Estimates of effect sizes (e.g. Cohen's *d*, Pearson's *r*), indicating how they were calculated

Our web collection on statistics for biologists contains articles on many of the points above.

Software and code

Policy information about availability of computer code

Data collection	Not applicable
Data analysis	Not applicable

For manuscripts utilizing custom algorithms or software that are central to the research but not yet described in published literature, software must be made available to editors/reviewers. We strongly encourage code deposition in a community repository (e.g. GitHub). See the Nature Research guidelines for submitting code & software for further information.

Data

Policy information about availability of data

All manuscripts must include a data availability statement. This statement should provide the following information, where applicable:

- Accession codes, unique identifiers, or web links for publicly available datasets
- A list of figures that have associated raw data
- A description of any restrictions on data availability

Sequence data for all the subjects studied in this work have been deposited in the European Genome-Phenome Archive (EGA) with the accession code EGAS00001003408 [https://www.ebi.ac.uk/ega/studies/EGAS00001003408].

Field-specific reporting

Please select the one below that is the best fit for your research. If you are not sure, read the appropriate sections before making your selection.

☒ Life sciences ☐ Behavioural & social sciences ☐ Ecological, evolutionary & environmental sciences

Life sciences study design

All studies must disclose on these points even when the disclosure is negative.

Sample size	A power analysis was performed to determine the number of non-NPC subjects required in the validation sample set given that we analysed 23 NPC patients from the screening cohort. Based on the means and standard deviations of the EBV DNA methylation scores of NPC patients and non-NPC subjects in the exploratory sample set, if we would reproduce the observed difference in the validation set at a significant level of 0.001 and a power of 0.999, at least 82 non-NPC subjects would be required.
Data exclusions	No data were excluded from the analyses.
Replication	The experiments were first performed in an exploratory cohort then validated in a validation cohort of 23 NPC samples (from the screening cohort), 14 NPC samples (from an external cohort) and 120 non-NPC samples (from the screening cohort). The samples in the validation cohort do not overlap with those from the exploratory cohort. The same samples were not analysed repeatedly.
Randomization	This is not a randomised study. The exploratory and validation cohorts consisted of samples of NPC and non-NPC subjects randomly selected from the published prospective screening study (Chan et al. NEJM 2017; 377:513-522).
Blinding	The exploratory and validation cohorts consisted of samples of NPC and non-NPC subjects randomly selected from the published prospective screening study (Chan et al. NEJM 2017; 377:513-522). Blinding is not feasible in the exploratory analysis as we need to define the cutoffs in the parameters based on the data from the NPC samples in the exploratory cohort. The investigators were blinded to the clinical status of the samples (NPC or non-NPC samples from the screening cohort) during data analysis in the validation phase.

Reporting for specific materials, systems and methods

We require information from authors about some types of materials, experimental systems and methods used in many studies. Here, indicate whether each material, system or method listed is relevant to your study. If you are not sure if a list item applies to your research, read the appropriate section before selecting a response.

Materials & experimental systems

n/a	Involved in the study	
☒	☐	Antibodies
☒	☐	Eukaryotic cell lines
☒	☐	Palaeontology
☒	☐	Animals and other organisms
☐	☒	Human research participants
☒	☐	Clinical data

Methods

n/a	Involved in the study	
☒	☐	ChIP-seq
☒	☐	Flow cytometry
☒	☐	MRI-based neuroimaging

Human research participants

Policy information about studies involving human research participants

Population characteristics	Archived plasma samples of patients with NPC and non-NPC subjects from the published prospective screening cohort were used for methylation analysis in the current study. In the screening study, we recruited subjects who were ethnically Chinese males aged between 40 and 62 years and did not show symptoms of NPC. We excluded subjects with history of cancer or autoimmune diseases and those who were receiving systemic glucocorticoids or immunosuppressive therapy.
Recruitment	The screening cohort is composed of 20,174 male subjects who were asymptomatic for nasopharyngeal carcinoma. We recruited these subjects through public health education sessions in Hong Kong between July 2013 and February 2016. There is no potential bias identified.
Ethics oversight	The study was approved by the Joint Chinese University of Hong Kong – Hospital Authority New Territories East Cluster Clinical Research Ethics Committee.

Note that full information on the approval of the study protocol must also be provided in the manuscript.
