# Supplementary material for: Methylation analysis of plasma DNA informs etiologies of Epstein-Barr virus-associated diseases
Source: Nat Commun. 2019 Jul 22;10:3256. doi: 10.1038/s41467-019-11226-5 (PMC6646310; doi:10.1038/s41467-019-11226-5)
Supplement: Supplementary file 6 — Description of Additional Supplementary Files [file 41467_2019_11226_MOESM6_ESM.docx]

**Title: Supplementary Data file 1**
**Description:** Individual methylation score over the 158 NPC-associated DMRs (methylation-based), proportion of EBV DNA reads (count-based) and EBV DNA size ratio (size-based) for all the cases in the exploratory and validation cohorts.

**Title: Supplementary Data file 2**
**Description:** A BED file with the EBV and human autosomal regions for the capture probe design.

**Title: Supplementary Data file 3**
**Description:** Quality control statistics of all the cases described in Figure 1 and the exploratory and validation cohorts.
